# Analysis of Factors Influencing Inpatient and Outpatient Satisfaction with the Chinese Military Health Service

**DOI:** 10.1371/journal.pone.0151234

**Published:** 2016-03-23

**Authors:** Yipeng Lv, Chen Xue, Yang Ge, Feng Ye, Xu Liu, Yuan Liu, Lulu Zhang

**Affiliations:** Department of Military Health Service, College of Military Health Service, Second Military Medical University, Shanghai 200433, China; Ottawa Hospital Research Institute, CANADA

## Abstract

**Background:**

Relatively few articles have focused on exploring factors influencing soldiers’ overall satisfaction and differences between inpatients’ and outpatients’ satisfaction, particularly in the Chinese army. Elucidating factors influencing military inpatient and outpatient care separately and analyzing their differences may provide more information for the healthsystem.

**Methods:**

The Revised China National Health Service Survey questionnaire was used in the survey. The questionnaire included 5 sections and 32 items concerning demographic, inpatient, and outpatient characteristics and perception variables for both inpatients and outpatients. Bivariate and multivariate techniques were used to reveal relationships between satisfaction and the variables assessed.

**Results:**

Outpatients’ and inpatients’ overall satisfaction rates were 19.0% and 18.5%, respectively. The strongest determinant of outpatients’ satisfaction was satisfaction with doctor’s communication regarding therapeutic regimen followed by length of military service, level of trust in medical staff, and disease severity. Determinants of inpatients’ satisfactionincludedstaff categories, satisfaction with environment, and satisfaction with medical quality.

**Conclusion:**

The factors influencing military outpatients’ satisfaction differed from those of inpatients. Exploring the causes of satisfaction and dissatisfaction with military health institutions is important in their fulfillment of their responsibility to maintain soldiers’ health.

## Introduction

Patients’ perception of healthcare isa critical indicator in measuring medical service quality. Medical disputes and violence against Chinese medical personnel often originate from a gap between care provision and patient expectationsconcerning ideal care.[1]Schoenfelderet al. posited thatdetermining the aspects of health services that influence satisfaction is essential in evaluating intervention effectiveness and improvingcarequality.[2]In 2009, The Chinese government started their medical and health service system reform to achieve more affordable national health care. A series of policies were enacted to improve the medical care and service system, public health system and the drug supply system. These factorscould be used to guide the healthsystem revolution. Inthe military health service, efficient improvement of medical care quality couldconsolidate soldiers’ fighting capacity. Therefore,exploration of the causes of satisfaction or dissatisfaction with military health institutions is of interest to the Military Ministry of Health in China.

Many researchershaveexplored the structure and factors influencing patient satisfaction and concluded that it is a multidimensional concept containing influential factorssuch as demographic characteristics, belief in care, waiting time,and information provision. Boudreaux argued that patients’ subjective experiences, rather than demographic and visit-related factors, are the most consistent determinants of satisfaction.[3] Similarly,Newsome and Wright (1999) reviewed 46 patientsatisfaction studies and found that the factors most commonly related to patient satisfaction were technical competence, interpersonal factors, convenience, costs, and facilities.[4]Bredartet al. posited that patients’ judgments regarding care quality are important for satisfactionwiththe technical quality of care, providers’ interpersonal skills, coordination, continuity, waiting times, availability, and physical environment.[5,6]Further, efficient doctor-patient communication was emphasized inprevious studies, while the environment was not. Predictors of satisfaction vary according tosetting,and improving key factors could advance healthcarequality and satisfaction.

Although various studieshave focused onexploring factors influencingcivilian satisfaction,relatively few have involvedmilitary personnel,particularlythose in the Chinese army. In china, military patients’ options are limited to specific health institutions while the citizen can receive civilian healthcare whenever and wherever they want which is of great difference. The military Soldiers’ health is directly related to troops’ daily training and fighting capacity. Consequently, military hospitals are important in guaranteeing military officers’and soldiers’ health. But just like the normal hospitals in china, military hospitals face serious challenge of uneven development. Grassroots medical institutions serve for great numbers of soldiers in China without advanced medical equipment and experienced medical staffs. Exploration of related satisfaction influencing factors is very meaningful. Adatabase search revealed only 7 articles describingsoldiers’ satisfaction with military healthcare and its influencing factors. Chaffin et al. andChisick et al.explored military soldiers’ satisfaction with dental hygiene providers[7–10], andZimlichman et al. and Bar-Dayan et al.examined military soldiers’ satisfaction withaprimary healthcare clinic[11–13]. However,they did not focus on differences in satisfaction between inpatients and outpatients. Considerable differencesin treatment measures, standards, time,and environment remain between inpatient and outpatient care. Patientsare concerned about different issueswhen receiving these2types of care. Elucidating factors influencing inpatient and outpatient care separately and analyzing their differences may provide more information for the health system. Military healthcare delivery shouldinclude services designed to map various healthcare needs and preferences. Weaimed to identify significant factors predicting inpatient and outpatient satisfaction inChinese military personnel and their relationship.

## Method

### Study design and setting

The survey was conducted in army establishments in Guangzhou and Hainan provinces. We recruitedmilitary personnel, ranging from soldiers to retired cadre, using random sampling. Inpatientswere soldierswho had received inpatient care the last yearand spent at least 1 night in hospital, while outpatientswere those who had received outpatientcare within thepreceding fortnight and did not stay in the hospital overnight. Weendeavored to minimize time-related recall bias because of these restrictive conditions. Patients with no cognitive impairment were eligible to participate.

All participants were assured that their responses would remain anonymous, and surveys did not include participant identifiers. Patients received consent forms, and participation was voluntary. For participants who were younger than 18 years of age,we obtained verbal consent for their participation from their guardians. Because the soldiers wererecruited throughout the country, it was almost impossible to obtain written consent during interviews; therefore, we spoke to minors’ guardians via telephone and recorded theirverbal consent. However,we were unable to contact the guardians of 8 soldiers who were younger than 18 years of age; therefore, we selected another 8 soldiers whose series numbers are next to them. All participants aged 18 years and older were asked to provide written informed consent prior to the initiation of the study. The study complied with all voluntary principles and was conducted in accordance with the Declaration of Helsinki. Ethical approval was granted by the ethics committee atthe Second Military Medical University. The consent procedure for the research wasalso approved by thisethics committee.

### Questionnaire design

Thequestionnaire used to explore soldiers’ satisfactionwas based on thatof the China National Health Service Surveyconducted by the China Ministry of Health (now the Health and Family Planning Commission) every 5 years for the past 25 years; results have beenapplied in Chinese health departments at all levels of scientific management and decisionmaking.[14,15]The questionnaire did not include insurance-related questions,asparticipantswere covered by the military healthcare system and were not required to pay for treatment. The questionnaire included5 sections and 32 items concerningdemographic,inpatient,and outpatient characteristics andperception variables for both inpatients and outpatients. In addition, the questionnaire also included a multiple–choice questionabout reasons of dissatisfaction.

#### Demographic characteristics

This section contained questions regarding sex,ethnicity, age,and length of military service.

#### Outpatient characteristics

This sectionincluded disease type,severity,illness time,number of days absent, number of treatments, and medical institution.

#### Inpatient characteristics

This sectionincluded operation situation,number of times hospitalized,time waited for hospitalization, time required to reach the medical institution,medical institution, hospitalization day, and discharge causes.

#### Perception variables

Thissectionincluded 4 questions concerning medical and service factors such as satisfaction with medical personnel’s explanation of conditions,satisfaction with doctor’s communication regarding therapeutic regimen, satisfaction with environment, level of trust in medical staff, and overall satisfaction. The inpatient subsection also included satisfaction with medical personnel’s attitudes and medical quality, while the outpatient subsectionincluded satisfaction with healthcare information.

Following careful screening, we distributed6,238 questionnaires;6,049were returned. Only 796 questionnaires were completed by participantswho had previously received inpatient or outpatient care, and 73of these were considered invalid. Therefore, we analyzed723 questionnaires including 521 and 248 containingoutpatient and inpatient data, respectively (some participants received both types of care). The Response rate is 12.0%.

### Statistical analysis

To simplify the data analysis,continuous variables were recoded into categorical variables. Descriptive statistics and frequencies were analyzed. Bivariate and multivariate techniques were used to reveal relationships between satisfaction and the variables assessed. SPSS 18.0 for windows was used for all analyses. Data regarding sex were discardedprior to analysis, as all but 7 soldiers were male. Overall satisfaction wasabinary variable;therefore,bivariate analysis involved a chi-square test. All associations were considered statistically significant at p<0.05. Due to the limited sample size,bivariate screening was performed to create sparse models with few degrees of freedom.[2]Multivariate analysis involved binarylogistic regression. Factors that were statistically significant in the bivariate analysis were analyzed usingbinary logistic regression to identify significant predictors of military soldiers’ satisfactionwith inpatient and outpatient care. In the logistic regression, missing data for the 6perception variables were substituted with average ratingsfor the respective questionnaire items,to ensure the largest possible dataset for multivariate analysis.[2]

## Results

The study sample consisted of 521 outpatients and 243 inpatients, all of whom completed the section concerning demographic characteristics. As shown in Table 1, in both inpatients and outpatients, Han was the most prevalent ethnicity, and most patients were aged 21–30years,while those older than 36 years comprised the smallest group. More than half of the soldiers had served in the army for less than 5 years. Most patients originated from middle and eastern regions. Regarding educational levels, in both groups,more than half of the soldiershad attended technical secondary or senior high school,andmanyhad attended university or junior college. Further,mostparticipants were sergeantsor conscripts.

**Table 1:** The relationship between the overall satisfaction and soldiers demographic characteristic of the outpatient and inpatient.

	Total	Satisfied Outpatient (%)	P value	Total	Satisfied Inpatient (%)	P value
ethnicity						
han	502	94(18.7%)	0.408	236	42(17.8%)	0.085
the other	19	5(26.3%)		7	3(42.9%)	
age						
36-	15	0	<0.0001***	6	0	0.119
31–35	51	8(15.7%)		34	6(17.6%)	
26–30	134	14(10.4%)		62	6(9.7%)	
21–25	240	47(19.6%)		110	24(21.8%)	
16–20	81	30(37.0%)		31	9(29.0%)	
length of military service						
16-	19	1(5.3%)	<0.0001***	11	1(9.1%)	0.100
11–15	61	8(13.1%)		38	6(15.8%)	
6–10	144	13(9.0%)		62	6(9.7%)	
1–5	297	77(25.9%)		132	32(24.2%)	
home address						
East	210	37(17.6%)	0.690	79	15(19.0%)	0.869
Middle	264	54(20.5%)		108	19(17.6%)	
West	47	8(17.0%)		56	11(19.6%)	
educational level						
graduate and junior college	210	30(14.3%)	0.024*	89	14(15.7%)	0.592
technical secondary school and senior high school	311	69(22.2%)		152	31(20.4%)	
junior high school and primarily school	0	0		2	0	
marital status						
unmarried	399	87(21.8%)	0.003**	181	40(22.1%)	0.018*
married	122	12(9.8%)		62	5(8.1%)	
staff category ^a^						
retired cadre	1	0	<0.0001***	2	1(50.0%)	<0.0001***
division level and above cadre	2	0		3	2(66.7%)	
regimental or below cadre levels	47	4(8.5%)		32	2(6.3%)	
Sergeant	323	46(14.2%)		140	15(10.7%)	
Conscript	143	47(32.9%)		62	23(37.1%)	
Cadetship	5	2(40.0%)		4	2(50.0%)	
workplace						
urban areas	195	44(22.6%)	0.420	82	23(28.0%)	0.083
rural areas	146	25(17.1%)		69	10(14.5%)	
Island	35	6(17.1%)		13	1(7.7%)	
Ship	145	24(16.6%)		79	11(13.9%)	

* P<0.05,

** P<0.01,

*** P<0.001

^a^. from Conscript to division level and above cadre, the military rank rises gradually. Cadetships are the students study in the military school and received the same medical treatment as conscripts. Retired cadre can enjoyed the best medical service in the Chinese military hospital.

Outpatients’ and inpatients’ overall satisfaction rateswere 19.0% and 18.5%, respectively. Reasonsfor soldiers’ dissatisfactionwere examined in the survey. The main reason for outpatient dissatisfaction was lack of medicine (33.4%), followed by poor service attitude (30.1%) and poor equipment (25.9%). In contrast, the main reason for inpatients’ dissatisfaction waspoor service attitude (40.3%), followed by lack of medicine (33.7%) and poor medical technology (32.9%).

Table 1 shows the relationship between overall satisfaction and soldiers’demographic characteristics. Age, length of military service, educational level, marital status, and staff category were related to outpatients’ overall satisfaction in the bivariate analyses. However,in inpatients, only marital status and staff category were related to overall satisfaction. In outpatients, soldiers aged 16–20years and those with 1–5 years of military service reported highersatisfaction levelsrelative tothose of other groups. Outpatientswith high educational levelswereless satisfied relative to other groups. Regarding staff category,the cadetshipcategory in outpatients and the division level or above cadrecategory in inpatientsshowed higher overall satisfactionlevels relative to those of other groups. Interestingly,in both outpatients and inpatients,married soldiers were less satisfied relative to unmarriedsoldiers.

Table 2 summarizes the relationships between soldiers’ satisfaction andillness status,outpatient care, and perception variables. Influenza, physical pain,disease severity,illness duration, medical institution, and subjective feeling influenced overall satisfaction. Suffering physical painreduced overall satisfaction, while patients with influenza reported higher satisfaction levels. Patients withmild diseasesor illnesses that had lasted for less than a fortnight reported higher satisfaction levels relative to those of other groups. “Receiving clinical care” is related to higher patient satisfaction rate. Patientswho responded“yes”or “very good”toperception items,such assatisfaction with healthcare information,satisfaction with doctor’s communication regarding therapeutic regimen, satisfaction with environment,and level of trust in medical staff,reported higher satisfaction levels relative to those of soldiers who responded “no.”

**Table 2:** The relationship between soldiers’ satisfaction and illness state, received outpatient care and perception variables.

	Total	Satisfied Outpatient (%)	P Value
type of diseases ^a^			
influenza	188	45(23.9%)	0.031*
Fever	56	10(17.9%)	0.817
Physical pain	175	23(13.1%)	0.015*
Stomach-ache	66	10(15.2%)	0.394
Training injury	73	12(16.4%)	0.547
Others	139	28(20.1%)	0.689
disease severity			
mild	107	34(31.8%)	0.001**
Moderate	308	50(16.2%)	
serious	106	15(14.2%)	
illness duration			
less than fortnight	267	66(24.7%)	0.001 **
acute disease happened two weeks before	51	7(13.7%)	
Chronic disease happened two weeks before	203	26(12.8%)	
The number of days absent			
more than 3 days	0	0	0.760
less than 3days	68	12(17.6%)	
Never	453	87(19.2%)	
the number of treatment			
more than 3 times	391	68(17.4%)	0.104
2 times	130	31(23.8%)	
1 time	0	0	
medical institution ^b^			
Clinics	312	69(22.1%)	0.041*
primary hospital	63	10(15.9%)	
secondary hospital	76	14(18.4%)	
tertiary hospital	70	6(8.6%)	
local hospital and others	0	0	
**Perception variables**			
Satisfaction with healthcare information			
yes	276	78(28.3%)	0.0001***
no	136	10(7.4%)	
not sure	109	11(10.1%)	
satisfaction with medical personnel’s explanation of condition			
very poor	32	1(3.1%)	0.0001***
Poor	41	2(4.9%)	
fair	297	35(11.8%)	
Good	92	31(33.7%)	
excellent	59	30(50.8%)	
satisfaction with doctor’s communication regarding therapeutic regimen			
very poor	38	1(2.6%)	0.0001***
Poor	55	3(5.5%)	
fair	273	35(12.8%)	
Good	99	29(29.3%)	
excellent	56	31(55.4%)	
satisfaction with environment			
very poor	23	1(4.3%)	0.0001***
Poor	41	0	
fair	300	42(14.0%)	
Good	98	28(28.6%)	
excellent	59	28(47.5%)	
the level of trust in medical staff			
very poor	23	1(4.3%)	0.0001***
Poor	30	1(3.3%)	
fair	277	32(11.6%)	
Good	147	40(27.2%)	
excellent	44	25(56.8%)	

* P<0.05,

** P<0.01,

*** P<0.001

^a^. all the p values of different types of disease are calculated separately about the comparison of those getting such disease and who did not.

^b^. From the clinic to the tertiary hospital, the medical resource and Diagnosis and treatment level growth greatly. Clinic means primary medical institutions without meeting the standard of hospital. The primary hospital is the hospitals focuses on the community or the grass-roots units providing the most basic medical service. The secondary hospital is the regional medical centre. The tertiary hospital is a kind of trans-regional hospital which has more medical resource and can provide advanced medical service. The local hospital and others means the hospitals beyond the military medical institutions.

Table 3 shows the relationships between inpatientsatisfaction and hospitalization-related factors andperception variables. None of the hospitalization-relatedfactorswere relatedto overall satisfaction. All of the items concerning patients’ subjective views of medical care influenced overall satisfaction.

**Table 3:** The relationship between soldiers satisfaction and the hospitalizations aspect, received inpatient care and perception variables.

	Total	Satisfied Inpatient (%)	P Value
operation situation			
Yes	148	26(17.6%)	0.785
No	95	18(18.9%)	
number of times hospitalized			
more than 1 time	42	10(23.8%)	0.291
1 times	201	34(16.9%)	
hospitalization day			
more than 7 days	177	34(19.2%)	0.637
3–7 days	45	6(13.3%)	
less than 3 days	21	4(19.0%)	
time waited for hospitalization			
more than 1 week	23	6(26.1%)	0.447
1 week	220	38(17.3%)	
the time required to research the medical institution			
more than 60 minutes	171	36(21.1%)	0.066
30–60 minutes	72	8(11.1%)	
0–30 minutes	0	0	
discharge causes			
bad treatment effect	5	1(20.0%)	0.351
poor treatment condition	3	0	
poor service attitude	5	0	
job demand	13	3(20.0%)	
self-feeling	16	1(6.3%)	
Cured	201	39(19.4%)	
medical institution			
Clinics	19	5(26.3%)	0.095
primary hospital	35	8(22.9%)	
secondary hospital	88	9(10.2%)	
tertiary hospital	53	9(17.0%)	
local hospital and others	48	13(27.1%)	
**Perception variables**			
satisfaction with medical personnel's explanation of condition			
very poor	7	2(28.6%)	0.005**
Poor	18	1(5.6%)	
Fair	126	16(12.7%)	
Good	56	10(17.9%)	
excellent	36	15(41.7%)	
satisfaction with doctor’s communication regarding therapeutic regimen			
very poor	7	2(28.6%)	0.004**
Poor	23	1(4.3%)	
Fair	131	17(13.0%)	
Good	48	11(22.9%)	
excellent	34	13(38.2%)	
satisfaction with environment			
very poor	9	1(11.1%)	0.001**
Poor	22	0	
Fair	129	19(14.7%)	
Good	49	11(22.4%)	
excellent	34	13(38.2%)	
the level of trust in medical staff			
very poor	8	1(12.5%)	0.007**
Poor	20	0	
Fair	113	15(13.3%)	
Good	72	19(26.4%)	
excellent	30	9(30.0%)	
satisfaction with medical personnel’s attitudes			
not satisfied	40	1(2.5%)	0.005**
satisfied	203	43(21.2%)	
satisfaction with medical quality			
not satisfied	35	1(2.9%)	0.010**
satisfied	208	44(21.2%)	

** P<0.01

Table 4 shows the results of the logistic regression analysis. The strongest determinant of outpatients’ overall satisfaction was satisfaction with doctor’s communication regarding therapeutic regimen, followed by length of military service, level of trust in medical staff,and disease severity. The determinants of inpatients’ overall satisfactionincludedstaff category,satisfaction with environment, and satisfaction with medical quality.

**Table 4:** Factors associated with overall outpatient satisfaction in the army after logistic regression analysis.

Variables	Odd ratio (95% confidence interval)	p Value
**Outpatient**		
length of military service	2.00(1.36–2.92)	0.0001
disease severity	0.63(0.43–0.94)	0.022
satisfaction with doctor’s communication regarding therapeutic regimen	2.09(1.50–2.90)	0.0001
the level of trust in the medical staffs	1.88(1.80–2.75)	0.001
**Inpatient**		
staff category		
regimental or below cadre levels	0.05(0.004–0.58)	0.017
sergeant	0.08 (0.01–0.69)	0.021
satisfaction with environment	1.69(1.13–2.53)	0.011
satisfaction with medical quality	7.59(1.24–61.16)	0.049

## Discussion

Inpatient and outpatient services differed with respect to treatment measures, standards, time, and environment. Outpatient care is a short-term medical service that does not require an overnight stay in hospital or a medical facility. In contrast, inpatient care involvescontinuity of care between patients and medical staff,in which inpatients’ perception of the environment and service process is valued. Outpatients’ and inpatients’ overall satisfaction rateswere19.0% and 18.5%, respectively. The overall satisfaction ratewas lower relative to those reported in other studies, suggesting that the care provided by the Chinese army does not fulfillsoldiers’ health needs. The literature review showed that soldiers’ overall satisfaction rates in previous studieswereapproximately 90%[16,17],while civilians’ satisfaction varied according to setting. Our survey showed that outpatientscomplainedmost about medicine shortages,while inpatients complainedabout the attitudes of medical staff.

As shown in the logistic regression analysis results,in both outpatients and inpatients,most demographic characteristics, such assex, age,ethnicity, home address, educational level, marital status, and workplace, did not remain significant in the logistic regression analysis. This result is consistent with those of previous studies, which showed that the influence of sociodemographic characteristics on patients’ satisfaction wasinconsistent. For example,with respect to education levels,Szycaet al. argued that more highly educated patientsdemonstrated lower expectations and greater satisfaction relative to those who had received less education[18], while Schulmeister et al. held the opposite view[19–23]. However,multivariate analysis identified an association between length of military service and outpatients’ overall satisfaction, while staff category influenced inpatients’ overall satisfaction. Specifically,soldiers in the regimental or below cadre and sergeant categories reported lower satisfaction levels relative to those of other groups. This phenomenon may have resulted from the considerable gap between soldiers’ expectations and the care that they received. Expectation has repeatedly emerged as a fundamental factor in satisfaction expression.[24–27] Patient satisfaction is related to perception of the benefits of care and the extent to which they meet patients’ expectations. Unlike the conscript and cadetship categories,these 2 groups represent military officials who shouldreceivesuperior and more comprehensive healthcare and insurance protection. However,relative to that of other officials, those in the cadre category received healthcare that was only marginally superior to that of soldiers, and grassroots medical institutions cannot meet their needs, causing embarrassment. Consequently, the healthcare and insurance protection received did not meet their expectations, which led to low satisfaction levels. The influence of length of military servicewas similar to that of staff category, which influenced soldiers’ perceptions ofmilitary health services. Soldiers achieved higher rank and received more advanced and comprehensive care with longer military service,which also led to higher expectations. Imbalanced health resource allocation is inappropriate and unsustainable and cannot maintain soldiers’ health. Additional health resources should be allocated to the grassroots army to improve soldiers’ perceptions of the health service.

Disease severity wasan influential factorinoverall patient satisfaction. Bredartet al. posited that patients’ judgments regarding care quality depend on theirphysical conditions.[5,6]Soldiers with serious diseases were constantlyunhappy, which mayhave led to pain and distrust of medical personnel. In contrast, patients are more familiar with mild diseases, such as influenza,and they demonstrate reasonable expectationsconcerning the disease and optimistic attitudes towards their treatment. These factors are all vital to overall satisfaction. It is worth noting that the medical institution factor was removed from the multivariate analysis. In China, hospitals were categorizedaccording to their functions, equipment, and technology levels. From clinics to tertiary hospitals, medical resources and diagnosis and treatment levelsincreasedconsiderably. Thereare strict medical service rules in place for treatment provided to soldiers, which stipulatethat theyare supposed to receive medical servicesfirst from primary military medical institutionsand then to advanced military hospitals. In some situations, they receive local,rather than military, medical services. The evaluation of medical services is always based on service quality, environment, and equipment. A numberof important factors that influence patient satisfaction, such as surroundings and medical service quality, have been analyzed individually. Therefore, the medical institution factor was eliminated from the multivariate analysis.

It is noteworthy that perception variables exerted a strong influence on overall satisfactionin both inpatients and outpatients. In the literature review, patient satisfaction was associated with decisionmaking,[28] clinicians’ communication,[29,30] treatment outcomes, patients’ expectations and therapeutic listening. In the present study, military outpatient valued trust and communication with medical staff. Previous studieshave emphasized the importance of communication.[22,29,31]This is congruent with our findings. To some extent, medical treatment is both a product provided by health institutions and a service enjoyed by patients. Consequently, patients valued both care quality and their perceptions regarding outpatient care. Efficient communication between medical personnel and patients increasestheir positivityregardingcare and establishes doctor-patient relationships, which can reduce patients’ anxiety.[32–34]This relationship was the foundation of patients’trust in medical staff, which couldhave influenced their satisfaction levels. In contrast, patients obtained information concerningtheir diseases and treatment via communication with medical staff, particularly in outpatient care. These results indicated that efficient communication improved patients’ perceptions of care andled to high satisfaction ratings. Unfortunately,this communication is neglected inhealthcare settings, evokingviolence and conflictbetween doctors and patients. In addition, patients’ trust in medical staff was also a factor influencing outpatients’ overall satisfaction. As mentioned previously, patients’ trust in medical staff and facilitiesaffects their attitudes and cooperation during treatment. All of these factors could promote patients’ recoveryand increase their satisfaction. Medical personnel should enhance communication with soldiers and provide clear treatment information. Most injured soldiers are young and far from their parents. Contact with doctors and nurses is more than a simple medical relationshipand constitutes a spiritual refuge for soldiers who have experienced intensive training and suffered emotional loneliness.

Hospitalization is highly stressful,and the relatedhealth service useconstitutesa long-term experience rather thansingle,short-term event. Multivariate analysis showed that environment and medical treatment quality strongly influencedinpatients’ overall satisfaction, which differedsignificantly from that of outpatients. Soldiers are inpatients only when seriously injured, which affects their training and faith. Undoubtedly,healthcare quality is important in patients’ perceptionsof care,particularlythose usinginpatient medical services. Traditionally,clinicians’ technical competence and mechanical precision are important factors in overall satisfaction assessment.[35]Unlike that of inpatient care, the quality of outpatient care is not observed immediately subsequent totreatment. However,inpatients are discharged only after they have receivedappropriate care and substantial symptom relief. Consequently, inpatient medical treatment quality was a primary factor when patientsappraised themedical services received. Furthermore, environment is also an important factor in soldiers’ overall satisfaction. To some extent,the hospital is type of hotel in which patients reside when they are ill. The comfort of their surroundingscould exert a strong influence on patients’ emotion and satisfaction, particularly for soldiers. In addition, the health institution’s environment reflectsthe scale and capacity of the department, which affects soldiers’ perceptionsofhospitalization. Clinics and primary hospitalsshould provide comfortableenvironments, which could offset nervousness and uneasiness in soldiers who are alone in hospital.

The study was subject tosome limitations. Because data werecollected from soldiersinHainan and Guangdong provinces,the representativeness of the findings is limited. Future research should include largersample sizes and armies from different military regions. Moreover,participantswererecruited from troops. If the studyhad been conducted in the hospital immediately following treatment, the sample could have been larger and recall bias minimized. Further, the data source was a self-report questionnaire. An objective scale and diagnosis or treatment information from hospitals’ patient databaseswould have been more precise. Despite these limitations,the study provided important information regarding inpatient and outpatient satisfaction in the Chinese army. It was the first study to explore factors influencing Chinese soldiers’ satisfaction with care,which couldcontribute to health system reform in China. Soldiers’ health was directly related to troops’ daily training and fighting capacity. Exploring the causes of satisfaction and dissatisfaction with military health institutions is important in thefulfillmentof their responsibility tomaintain soldiers’ health.

## Conclusion

Soldiers’ health status is directly related to troops’ daily training and fighting capacity. Exploring the causes of satisfaction or dissatisfaction with military health institutions is important in ensuring that military health institutions fulfill their responsibility to maintain soldiers’ health. Bivariate and multivariate techniques were used to reveal the relationship between satisfaction and assessed variables. Outpatient satisfaction was related to doctor-patient communication concerning the therapeutic regimen, length of military service, levels of trust in medical staff, and the severity of disease. In contrast, inpatient satisfaction was associated with staff category, satisfaction with the environment, and satisfaction with medical quality. Health resource location reform, communication enhancement, and environment improvement are essential in increasing satisfaction.
